# Photosensized Controlling Benzyl Methacrylate-Based Matrix Enhanced Eu^3+^ Narrow-Band Emission for Fluorescence Applications

**DOI:** 10.3390/ijms13033718

**Published:** 2012-03-21

**Authors:** Jiann-Fong Lee, Hsuen-Li Chen, Geneh-Siang Lee, Shao-Chin Tseng, Mei-Hsiang Lin, Wen-Bin Liau

**Affiliations:** 1 Department of Materials Science and Engineering, National Taiwan University, No. 1, Sec. 4, Roosevelt Road, Taipei 10617, Taiwan; E-Mails: douya2@yahoo.com.tw (J.-F.L.); hsenlichen@ntu.edu.tw (H.-L.C.); d96527023@ntu.edu.tw (S.-C.T.); 2 Instrumentation Center, National Taiwan University, No. 1, Sec. 4, Roosevelt Road, Taipei 10617, Taiwan; E-Mail: ghlee@ntu.edu.tw; 3 College of Pharmacy, School of Pharmacy, Taipei Medical University, Taipei 11031, Taiwan; E-Mail: mhl00001@tmu.edu.tw

**Keywords:** europium complex, UV-curing, optical tuning, metal-ion chelating, fluorescence detection

## Abstract

This study synthesized a europium (Eu^3+^) complex Eu(DBM)_3_Cl-MIP (DBM = dibenzoyl methane; Cl-MIP = 2-(2-chlorophenyl)-1-methyl-1*H*-imidazo[4,5-f][1,10]phenanthroline) dispersed in a benzyl methacrylate (BMA) monomer and treated with ultraviolet (UV) light for polymerization. Spectral results showed that the europium complex containing an antenna, Cl-MIP, which had higher triplet energy into the Eu^3+^ energy level, was an energetically enhanced europium emission. Typical stacking behaviors of π–π interactions between the ligands and the Eu^3+^-ion were analyzed using single crystal X-ray diffraction. Regarding the luminescence performance of this europium composite, the ligand/defect emission was suppressed by dispersion in a poly-BMA (PBMA) matrix. The underlying mechanism of the effective enhancement of the pure Eu^3+^ emission was attributed to the combined effects of structural modifications, defect emissions, and carrier charge transfer. Fluorescence spectra were compared to the composite of optimized Eu3+ emission where they were subsequently chelated to four metal ions via carboxylate groups on the BMA unit. The optical enhanced europium composite clearly demonstrated highly efficient optical responses and is, therefore a promising application as an optical detection material.

## 1. Introduction

Lanthanide-chelate (LC) emissions have widely attracted intensive research because of their unique optical emissions spanning a wide wavelength range from ultraviolet (UV)-visible to near-infrared regions [1–8]. Our focus was on organic europium-chelate (EC) emissions that have a narrow peak profile and stable luminescence. Such emissions are an ideal platform for DNA binding, bioanalyte and chemical fluorescence, which operate at highly selective wavelengths and demonstrate excellent optical stability [9–12]. Obtaining a pure emission color from small organic molecules and conjugated polymers is difficult because their emission bands typically have a full width at half maximum (FWHM) of 50 to 200 nm, and this larger band structure affects the accuracy of fluorescence responses at certain wavelengths. As is known, thin-film chromophores exhibit a substantial number of photoexcited carriers, which may be trapped by impurities, oxygen, or structural defects, causing poor fluorescent quantum efficiencies [13–15]. Furthermore, aggregation of these chromophores with strong π–π interactions can induce the carrier trapped in solid states [16]. To overcome these obstacles and obtain pure Eu^3+^ emissions with high absorption sections from its ligands, several methods have been employed to improve the optical properties of ECs. These include using chemical modification, a polymer/inorganic luminescent matrix, or doping with other lanthanide ions which would improve their essence of weak *f*–*f* emissions [17,18]. Covalent and non-covalent energy transfer pathways have been used to continue developing new EC designs. Chemical modifications of these organic europium complexes have caused remarkable amplification of the ligand’s triplet energy through exciton-interactions, thereby enhancing EC fluorescence [19,20]. However, despite these polymers or inorganic ECs being incorporated in host-guest systems, challenging obstacles remain, such as agglomerates and leakage of either ions or polymer ionomers, which have always constituted a concern in EC applications [7–9,21].

Many studies have tried to reduce such optical losses of the ECs by accessing coordinated or anionic ligands (most of which are β-diketones) of ternary europium, such as pyridylcyclotetramine-based [22], Eu(tta)_3_dpbt [23], and coumarin-based europium [24]; the quantum fluorescent efficiencies (QEs) of which have been reported to achieve between 30% and 60%. We report a europium complex containing a Cl-MIP as a coordinated ligand with an excellent QE achieved in solution and in film. The resultant EC complex also showed several extra advantages, such as improved emission lifetime, facile synthesis/purification, and easy tuning of optical responses from their morphologies. Using the host-guest strategy to prepare EC-polymers can be categorized into two main types. As is known, the first type allows access of covalent or non-covalent EC complexes to a luminescent polymer, such as *p-*(*N*-vinylcarbazole) (PVK) or *p*-(phenylene vinylenes) (PPVs) [2,6,16]. However, these rigid polymers are intended to be partially soluble in EC solutions, which cause incomplete ligand-to-metal energy transfer to EC emissions. The second approach for preparing EC-polymers relies on well-controlled adjacent chemical microstructures from radical polymerization, such as thermo- and photo-polymerization to meet the optimized properties. We directly synthesized EC-composites from acrylate-based polymers in the presence of suitable monomers. This polymer matrix herein showed effective aggregation suppression of ECs by controlling the dispersive medium. However, these approaches are generally complex and difficult to control. Therefore, determining how to obtain pure EC emissions without ligand/defect emissions and to achieve a high QE in a single layer would have potential for actual applications of ECs.

**Scheme 1:**
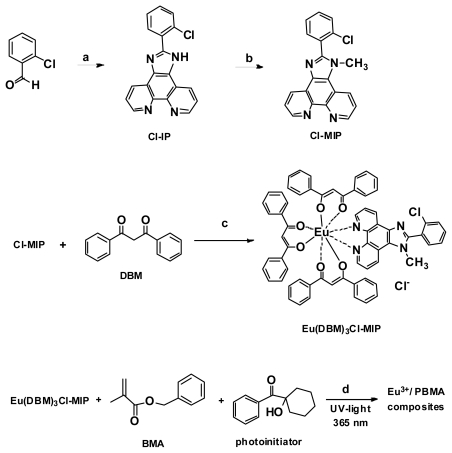
Synthetic illustration of the preparation of the ligand 2-(2-chlorophenyl)-1-methyl-1*H*-imidazo[4,5-f][1,10] phenanthroline (Cl-MIP), Eu^3+^compound (Eu(DBM)_3_Cl-MIP), and Eu^3+^/poly-BMA (PBMA) composite films by conducting a photo-polymerization process. Conditions included: (**a**) NH_4_OAc, phenanthracenedione, glacial HOAc, reflux for 2 h (61%); (**b**) NaH, CH_3_I, DMF at room temperature for 18 h (85%); and (**c**) 1 N NaOH (aq), EuCl_3_.6H_2_O, anhydrous EtOH at 50 °C for 5 h, pH~7 (67%); (**d**) The test sample was covered with PET film, and then exposed to ultraviolet light for 300 s.

We chose a host-guest approach to develop polymer-chromophore systems. PBMA matrix (the structure of which is shown in Scheme 1) is amorphous, has low reflectance, as well as metal/ion chelating stability [10,25–26], and is a class of optically transparent, UV-curable polymers. This precursor monomer provides excellent solubility and ionic stability, which should be advantageous for high-quality film fabrication after photo-polymerization. There are numerous materials and methods for rapidly obtained shapes and there is an efficient way to produce a homogeneous thin film with one UV-cured acrylate. Materials with various morphologies can be designed quickly [27–28], and the matrix with extensive carboxylate groups has enhanced photoluminescence stability [18]. At the outset, we attempted to solve the purification problems and found that organic impurities, moisture, and air species that dissolved in the precursor mixture could be eliminated through argon purging and freeze-thaw cycles [29,30]. Besides, these procedures improved the EC emissive pathways. The detected PL corresponding to the strongest peak of the Eu^3+^ signal (^5^D_0_→^7^F_2,_ 611 nm) could be enhanced by approximately 2.1-fold of that of the pristine Eu^3+^ complex. The ratio between the pure Eu^3+^ and defect emissions was more than two orders of magnitude higher, implying that this optical alignment could successfully increase the EC signal. Some transition metal ions are known to be efficient quenchers of the emissions of europium complexes [10]. With regard to this aspect, the fluorescence spectra of metal ion-chelated and optical modified europium composites were simultaneously compared. They were assisted to clarify the confines of their energy transfer between the europium emissive levels. The structural properties and photophysical behaviors of composites of a europium complex bound to PBMA were extensively demonstrated.

## 2. Results and Discussion

### 2.1. Studies of Ligand Properties

To verify the underlying mechanism for Eu^3+^ PL enhancement, an efficient ligand, named Chloro-phenylmethyl imidazo-phenanthroline (Cl-MIP), was used in this study and subjected to chemical modification. Mondel *et al*. and other previous studies have indicated that strong π–π interactions between neighboring imidazo-phenanthroline (IP) and aromatic units in aryl-*σ*-IP (AIP) chromophores resulted in a rigid structure and high electron mobility [31]. Nevertheless, the random arrangement of AIP molecules, usually with a strong aggregation tendency, largely affects the optical extraction performance of AIP; such as with charge dynamics, luminescence yield, and absorption profiles; similar results were shown in our related compounds [26]. An effective molecular design can obtain dynamic advantages of AIP and control its desired aggregation features. Cl-MIP served as an antenna ligand of the europium complex. This ligand was expected to have greater triplet energy transport away from its singlet pathway. The efficiency of triplet electronic energy transfer (EET) was clearly confirmed according to its parameters of molar absorption coefficient, molecular interaction and QE value.

### 2.2. Single Crystals of Cl-MIP and Eu(DBM)_3_Cl-MIP

**Chart 1:**
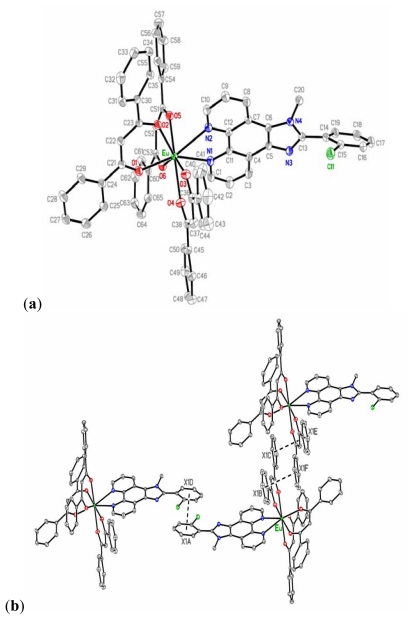
(**a**) Molecular structure of Eu(DBM)_3_Cl-MIP, showing the atom-labeling scheme and thermal ellipsoids; (**b**) π–π interactions of the Eu(DBM)_3_Cl-MIP complex. Dashed lines represent π–π interactions.

Crystal types and molecular packing of EC systems are critical in understanding the molecular interactive effects, including single crystals, packing diagrams, and hydrogen bond analyses; related data are summarized in Tables S1–S3 (electronic supplemental information (ESI^†^)). In individual molecular analyses, the free ligand of Cl-MIP consisted of 1-methyl-1*H*-imidazo[4,5,f] [1,10]phenanthroline and a 2-chlorophenyl ring, as shown in Figure S1 (ESI^†^). The dihedral angle between the IP unit and the phenyl ring was 64.15(4)°. The solvent molecule of CH_2_Cl_2_ was co-crystallized with the Cl-MIP molecule. The crystal structure was stabilized by a series of hydrogen bonds between the CH_2_Cl_2_ and Cl-MIP molecules. The packing diagram of Cl-MIP is shown in Figure S2. The molecular structure of Eu(DBM)_3_Cl-MIP is shown in Chart 1a. The central ion of Eu(III) is eight-coordinated by six oxygen atoms of three bidentate DBM anions and two nitrogen atoms from a bidentate Cl-MIP ligand, forming a distorted square antiprism. The average Eu-O bond distance was 2.318(2) Å, and the mean Eu-N bond distance was 2.596(2) Å. The dihedral angle between the imidazo-phenanthroline unit and the phenyl ring was 51.51(8)°, and was smaller than the free ligand at 64.15(4)°. Two phenyl rings from a DBM anion between the adjacent molecules were nearly parallel, with inter-centroid distances of 3.731(2) and 3.731(2) Å; and the shortest inter-ring distances were 3.447(1) and 3.402(1) Å. The results revealed π–π interactions. An additional π–π stacking was seen between two phenyl rings from adjacent Cl-MIP ligands where the inter-centroid distance was 3.908(2) Å, and the shortest inter-ring distance was 3.689(2) Å. These π–π interactions are shown in Chart 1b.

Cl-MIP and Eu(DBM)_3_Cl-MIP with strong π–π stacking between the aromatic units, were shown to have good electron transport and high triplet energy as found by performing spectral analyses [25]. The aggregation behaviors of these two molecules not only led to a red shift of their emission spectra, but also resulted in deterioration of the solid-state optical performance. In view of this, the PBMA selected to act as an isolative and dispersive matrix was studied [18,22]. Aggregate suppression between the aromatic ligands was caused by increased steric congestion [25]. Where a heavier halogen atom was replaced by a hydrogen atom in the 2-position of the phenyl ring, the europium complex obtained extra carrier dynamics benefits, allowing a greater amount of excited energy from intersystem crossing (ISC) relax to the triplet state by the heavy ion effect [32]. Molecular studies indicated that, effective energy-transfer distance and polar aggregation can be reduced when the IP unit bears a methyl substitute [22,33].

### 2.3. Photophysical Properties of Europium/PBMA Composites

**Figure 1:**
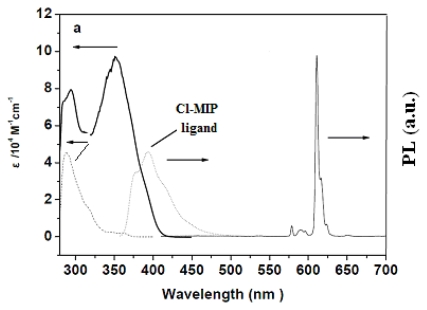

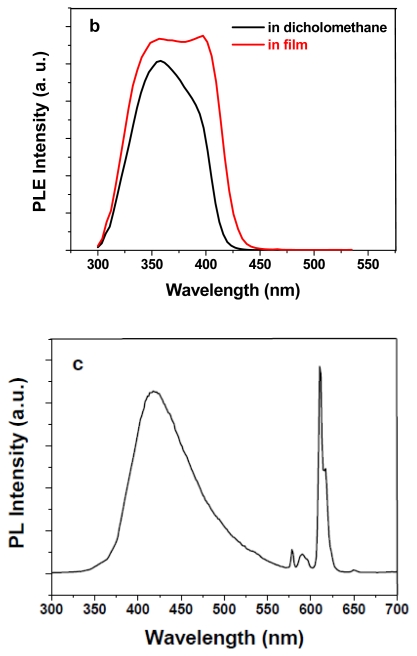
(**a**) Absorption and Photoluminescence (PL) spectra of Cl-MIP and Eu(DBM)_3_Cl-MIP; (**b**) Photoluminescence excitation spectrum of Eu(DBM)_3_Cl-MIP (in CH_2_Cl_2_, 2 × 10^−5^ M); (**c**) Photoluminescence spectrum of Eu(DBM)_3_Cl-MIP with the benzyl methacrylate (BMA) matrix at a 1.0 wt% level, after ultraviolet-lamp curing for 360 s.

**Figure 2:**
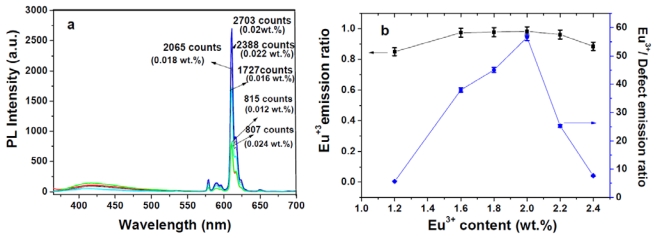
(**a**) Intensities of the photoluminescence spectra of the europium/PBMA composite with different Eu^3+^ contents; (**b**) Dependence of the Eu^3+^ emission ratio and Eu^3+^/defect emission ratio of the Eu^3+^ content.

**Figure 3:**
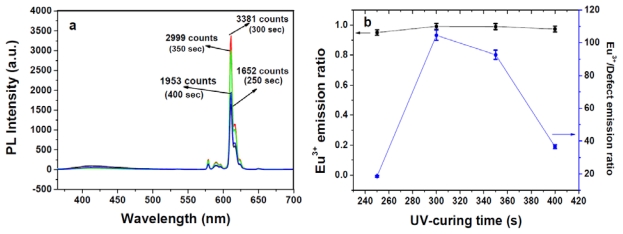
(**a**) Intensities of the photoluminescence spectra of the europium/PBMA composite with different ultraviolet (UV)-curing time; (**b**) Dependence of the europium emission ratio and Eu^3+^/defect on the BMA UV-curing time.

Figure 1a,b, as well as the inset of 1b, shows the absorption, PL, and photoluminescent excitation (PLE) spectra of Eu^3+^ transition levels. The FWHM value of the Eu^3+^ emission spectrum in the solid state was approximately 4 nm, and the intense emission line at 611 nm was from ^5^D_0_→^7^F_2_. The molar absorption coefficient (*ɛ*) values of the absorption spectrum exhibited by this new europium complex reached *λ*_max_ (*ɛ*) = 352 (974,000) and 294 nm (72,400) in dichloromethane; the high absorption values of Cl-MIP can be competitive to similar AIPs [23,24]. This also demonstrated that sensitization of the europium complex through its singlet pathway can easily transport ligand-absorption energy levels to triplet states [34]. The optical absorption, photoluminescence (PL), quantum yield, electrochemical data, and thermal properties are summarized in Table 1. The photoluminescent excited spectrum showed a broad band at approximately 375–420 nm, which was attributed to the ligand’s π–π overlap, inducing a charge-transfer of the Eu^3+^ complex. The monitored diluted-state PL was excitation at 357 nm (Figure 1b). Figure 1c shows a broad band at approximately 417 nm, attributed to a ligand emission at the 1.0 wt% level of Eu^3+^ and based on the weight of PBMA. At lower europium content there was a large number of excited carriers trapped in the PBMA matrix, which conducted a poor Eu^3+^ PL signal. In this work, we adjusted the concentration of Eu^3+^ and the light-irradiation time to determine the maximal fluorescent response. Figure 2a shows the PL spectra of Eu^3+^/PBMA composites with various Eu^3+^ contents. The spectrum was the result of excitation by 365 nm radiation from a 400-W TP UV-lamp for 360 s. When the europium content was in the range of 1.0–2.0 wt%, it exhibited weak PL signals (611 nm) and a relatively strong defect emission that belongs to ligands detected at approximately 417 nm. When the Eu^3+^ content achieved a value of 2.0 wt%, a large enhancement of the Eu^3+^ emissions (611 nm, ^5^D_0_→^7^F_2_) was recorded. Simultaneously, the defect emission was reduced. Figure 2a demonstrates a threshold concentration level of the EC carrier that was irreversibly transported at its emissive levels. Here, the PL intensity ratio between the Eu^3+^ narrow-band and defect contents exceeded > 2.0 wt%, the Eu^3+^ emissions dramatically decreased, and the defect emission ratio exhibited a rapid increase, while a fluorescence-quenching mechanism rapidly increased. The UV-curing duration was simultaneously controlled to detect PL signals at 2 wt% Eu^3+^. This further purified the optical signals of PL intensity that appeared in the Eu^3+^/defect emission ratio. Thus, the PL intensity ratio of Eu^3+^ and the defect emission was amplified by up to 104-fold, as shown in Figure 3a,b.

### 2.4. Aggregated Nature of Europium/PBMA Composites

**Figure 4:**
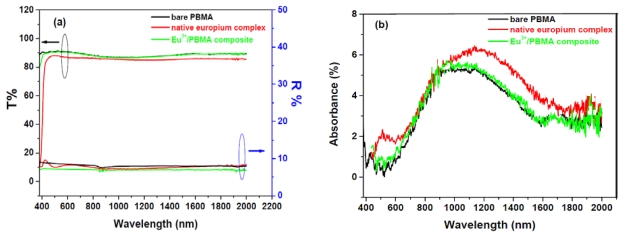
Optical transmittance, reflectance (**a**) and absorbance (**b**) spectra in the thin-film state. The test samples were bare PBMA (black-line), PRISTINE Eu(DBM)_3_Cl-MIP (red-line), and a Eu^3+^/PBMA composite (green-line), with an average thickness of ~22 μm and a scan range of 0.4~2.0 μm.

By contrast, we attempted to understand how the fluorescence efficiency is influenced by structural properties; therefore, we tuned the adjacent guest/host microstructures. Compared to different UV-irradiation times, we clearly identified that the maximal ligand emission appeared at approximately 417 nm, implying that PL responses of the carrier pathways or microstructures of the EC complex had changed. The triplet energy transfer of ECs is highly dependent on the Eu-ligand distance and outer microstructure of the PBMA matrix [36–39]. Controlling the coordinated environments and luminescence mechanism of the EC complex by conducting certain procedures can achieve high performance of EC emissions. The maximal PL intensity of EC composites appeared at approximately 300 s when comparing different UV-curing times. The microstructure enhancement seemed to be improved, because most excited carriers were trapped at the ligand level. Spectral reflectance and transmittance were measured to examine the optical profiles of this EC composite with a scan range of 0.4–2.0 μm. Figure 4a,b shows the optical properties of each constituent component. PBMA and the europium/PBMA composite displayed excellent optical transmittance (>88%) and lower reflectance (<8.2% for PBMA and <7.9% for the europium/PBMA composite in the visible region with maximal values of 7.6% for PBMA and 5.6% for the europium/PBMA composite), indicating that the europium/PBMA composite system can be regarded as a high-transmittance material in the range of visible to near-IR radiation. This also suggests that the EC composite is capable of acting as a fluorescent signal for detection in this wavelength region [11,14]. As expected, the pristine Eu^3+^ complex showed a red-shifted absorption band at 420–600 nm and exhibited poorer transmittance in the overall range than that of neat PBMA and the europium/PBMA composites. Figure 4b shows the absorbance of the constituent components in the same wavelength range, where R values were calculated by the equation of A % (absorbance) = (1 − R − T) × 100%.

### 2.5. Emissive Transitional States of Europium/PBMA Composite

**Figure 5:**
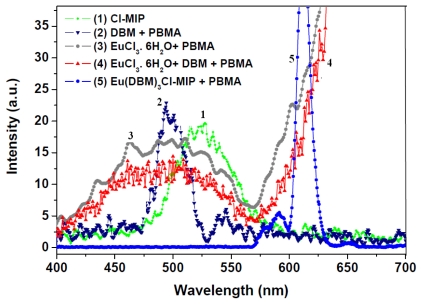
Phosphorescence spectra of (1) Cl-MIP (green-line); (2) DBM in PBMA (navy-line); (3) EuCl_3_.6H20 in PBMA (grey-line); (4) EuCl_3_.6 H_2_O + DBM in PBMA (red-line); and (5) Eu(DBM)_3_Cl-MIP (blue-line), λ_ex_ = 375 nm.

To evaluate this new EC on the energy transfer effects in the PBMA matrix, we further probed the donor: DBM, Cl-MIP; and the acceptor: the europium ion to detect their triplet state energy levels; the results are shown in Figure 5. These phosphorescence spectra use 375 nm as the excitation source. By comparison, Cl-MIP has a broad emission at 525 nm; DBM in a PBMA system (line-2) shows a broad phosphorescence band (λ_max_ = 500 nm) corresponding to singlet state emission. The phosphorescent results are consistent with an earlier report [18]. In this system, we also discovered a weak band, possibly attributed to a PBMA emission of the triplet state, which appeared at approximately 520 nm. Emission at this wavelength has also been observed in PBMA-DBM mixtures doped with Eu^3+^ (lines 3 and 4). The highest Eu^3+^ phosphorescence emission is observed in the Eu(DBM)_3_Cl-MIP-PBMA (line-5) and herein there is no indication of DBM, Cl-MIP, or PBMA triplet state emission. As is known, the excited Cl-MIP and DBM photons undergo a nonradiative transition to their longer lived triplet state.

### 2.6. Emission Lifetime Analyses of Eu^3+^/PBMA Composites

We further probed the interaction between the EC and adjacent microstructures by monitoring the emission decay, using a 375 nm laser pulse as the excitation source. This decay curve fitting showed a monoexponetial kinetic at ^5^D_0_→^7^F_2_ transition, and exhibits characteristic decay of 220 μs. For the Eu(Phen)_3_.H_2_O/PMMA system, the decay curve of the Eu^3+^ ion in the presence of DBM can be fitted to a multiexponential function to yield a longer decay of 670 μs [18]. An increase in the decay flourescence of Eu^3+^ appeared in the range of 670 to 740 μs. Here, these EC decay curves are effectively fitted by biexponential decay kinetics, which can be expressed in Equation 1, and we determined their average lifetime ( *t* ) using Equation 2.

(1) $$\text{F}(\text{t})=a_{1}\text{exp}(-\text{t}/\tau_{1})+a_{2}\text{exp}(-\text{t}/\tau_{2})$$

(2) $$\langle t\rangle =\sum a_{i}{\tau_{i}}^{2}/\sum a_{i}\tau_{i}$$

**Figure 6:**
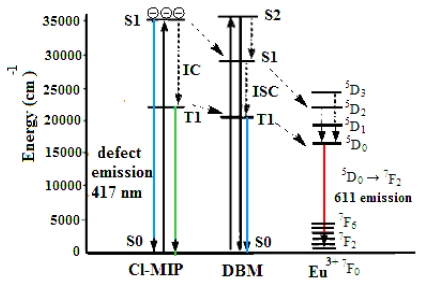
Carrier transfer process of Eu^3+^/BMA composites with and without emissive defects in Eu^3+^ resonance energy levels. Internal conversion (IC); intersystem crossing process (ISC).

By substituting the values of *a_1_*, *a_2_*, *τ*_1_, and *τ*_2_ in Equation 2, we obtained the average lifetime of each EC, as summarized in Table 2. The emission lifetime among these three EC compositions, doped in PBMA, in ascending order is: EC doped in PBMA with argon purging and a freeze-thaw cycle (730 μs) > EC doped in PBMA composite (651 μs) > neat EC system (565 μs). The observed emission lifetime indicates that the ligands interacting with the PBMA matrix results in energy transfer enhanced by the ligand’s effective shielding. Thus, the sequence of the energy transfer among the ligands is in solely PL intensity order of their emission lifetime. Figure 6 illustrates the carrier charge-transfer process used to interpret the Eu^3+^ photo-induced emission mechanism. After photoexcitation and generation of the carrier, the excited electrons were non-radiatively transferred to L1; and the lowest energy of Eu^3+^ surrounded the ligands, which subsequently relaxed to 1 the triplet state of T1 before beginning the intersystem cross-transfer to Eu^3+^ emission levels. Under lower carrier concentrations of the europium complex or an inadequate UV-irradiation dose, the carriers were trapped in the ligand molecules and were unable to penetrate and diffuse to the center of the Eu^3+^-ion energy levels. The photoexcited electrons, herein confined to the ligand state, created another transition pathway from the lowest level of the singlet state.

### 2.7. Controlling Optimized Fluorescence Response of Europium/PBMA Composite

**Figure 7:**
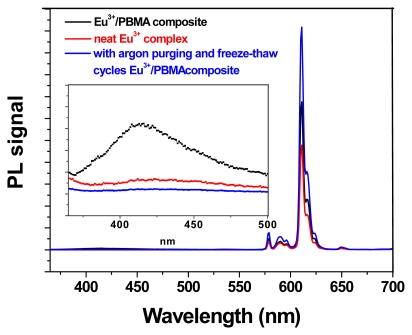
Photoluminescence spectra of the Eu^3+^/PBMA composite and neat Eu(DBM)_3_Cl-MIP under identical conditions. Ligand emission signals indicate an amplified scale of ~417 nm, as shown in the inset.

We adjusted the Eu^3+^ concentration and the light-irradiation duration to increase the efficiency of the EC fluorescence. This decreased the probability of photoexcited electrons being trapped, and led to an enhancement of europium emissions. In our proposed mechanism for PL enhancement, the existence of a carrier confined to the ligand-state performs a vital function. Usually, the carrier coupling from the ligand state can easily transfer to Eu^3+^ energy levels because of the non-trapped photoexcited electrons in the europium complex. Under this condition, pure Eu^3+^ PL signals were amplified by adjusting the concentration of europium and the UV-curing duration. To confirm this prediction, a europium/PBMA sample was optimized. The measured PL spectra showed considerable enhancement of europium emissions, but substantial ligand emissions still remained. Dissolved impurities in the precursor mixture trapped carriers in the defect state, leading to the ligand’s emission. Through argon purging and a freeze-thaw cycling technique to remove impurities from the precursor, the pure europium PL signal was enhanced. Figure 7 shows the europium/PBMA composite at 2.0 wt% of Eu^3+^, after UV-lamp curing for 300 s, for which the strongest peak of the pure Eu^3+^ PL signal (^5^D_0_→^7^F_2_, 611 nm) was enhanced by approximately 2.1-fold of that of the pristine Eu(DBM)_3_Cl-MIP, as shown in the inset figure. The ratio between pure Eu^3+^ and the defective emissions were more than two orders of magnitude higher, implying that this optical alignment successfully enhanced the Eu^3+^ PL signal.

### 2.8. Energy Transfer to Metal Ions within Europium/PBMA Composites

High performance of polymer-based europium composite greatly depends on the synthesis methods structural nature, and morphological result. In this article, the optimized chemical, micro-structural and optical controlled europium composites were extensively demonstrated. These approaches provided more efficient enhancement of the energy transfer between the Eu^3+^ emissive levels. The charge/energy transfer interactions between europium complexes and metal ions were investigated using fluorescence quenching spectroscopy [10,40]. In a feasible approach, the photostability of a europium composite accessing metal ion can be used to evaluate the europium material’s optical sensitivity by metal ion-chelating in the matrix, altering electron transfer from the ligands triplet states into the Eu^3+^ emissive levels.

**Figure 8:**
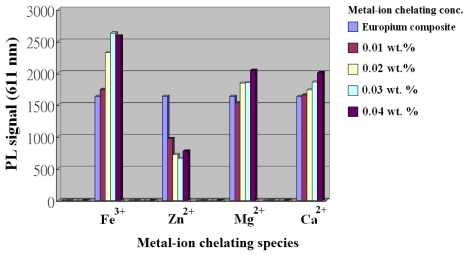
PL signal variations of Fe^3+^, Zn^2+^, Ca^2+^, and Mg^2+^ ions by weight concentration in Eu^3+^/PBMA and ionic contents obtained from those ions as a solution with a 0.0243 g/0.5 g BMA mixture. The fluorescence signals, *in situ* recording of four metal ions binding to the Eu^3+^/PBMA composite at a scan rate of 240 nm/min and excitation at 360 nm.

We used a series of simple metal ions, including Fe^3+^, Zn^2+^, Ca^2+^, and Mg^2+^ chelated to the carboxylate group on the BMA unit, which provided useful insight into the behavior of these photoinduced carriers at trace levels. Figure 8 shows that pristine europium fluorescence was synthesized following the optimized conditions, so that the europium composite with the highest fluorescent intensity at ^5^D_0_→^7^F_2_ transition (611 nm) was the referenced sample; other europium samples were prepared corresponding to europium/PBMA contents of 0~0.04 wt%. Results showed that PL signals occurred at different directions in species of four metal ions. The presence of Zn^2+^ was inefficient in quenching europium emission in the specific range, which may be attributed to highly diminished triplet carriers between the intercrossing and europium energy levels. Next, small PL increases that appeared in the Ca^2+^ and Mg^2+^ ions corresponded to contents of a certain wt%, which showed no significant change on europium emission and PLE spectra. The presence of Ca^2+^ and Mg^2+^ ions seem to give weak interactions with europium complex in this region, therefore, the optical feature was not investigated further. As for the contents at 0–0.04 wt% for Fe^3+^, the PL signals were significantly increased in this region. The PL increased as the Fe^3+^ proportion by weight of europium composite increased to a maximum of ~60% level, the percentage increase was relative to the referenced sample. This may be attributed to stronger attachments linking the europium and polymer pathways by Fe^3+^, allowing more carrier populations into the Eu^3+^ emissive levels [41]. Consequently, we investigated its absorption and PLE spectra demonstrated that no new charge-transport or ligand bands occurred, indicating that Fe^3+^ can largely enhance the PL signal of europium-chelated at the triplet state. This europium material will be helpful to achieve highly fluorescent efficiency in optical detections.

## 3. Experimental Section

### 3.1. Materials

Benzyl methacrylate (BMA) (Alfa Aesar, >99%) was passed through basic alumina to remove impurities, and the photoinitiator, (1-hydroxycyclohexyl) (phenyl) methanone (98%, Dupont Chemicals), was crystallized from ethyl acetate (EA) (3 mL/g). Dibenzoyl methane (DBM, >98%) was crystallized from toluene (2.5 mL/g). Most synthetic chemicals were purchased from Sigma-Aldrich or Acros Organics and were used as received. All solvents used in the experiments were purified following laboratory chemical procedures. Synthetic procedures and the yields of the ligand (Cl-MIP) and Eu(DBM)_3_Cl-MIP are shown in Scheme 1.

### 3.2. Methods

^1^H nuclear magnetic resonance (NMR) and ^13^C NMR spectra were recorded using a Bruker DMX-500 FT-NMR spectrometer. Chemical shifts were recorded in parts per million (ppm) downfield from that of the Me_4_Si standard. High-resolution electrospray ionization (ESI) mass spectrometric (HRMS (ESI)) data were obtained by a JEOL JMS-700 spectrometer (Tokyo, Japan). Photoluminescence (PL) measurements were performed with a Hitachi F-4500 fluorescence spectrophotometer (Japan) equipped with a 150-W xenon lamp. A solid-state quantum yield was determined by an F-7000 Fluorescence Spectrophotometer (Hitachi, Japan) using an integrating sphere (part No. 5J0–0148) to observe the standard sample and the corresponding europium complex. Crystallographic analyses of the ligand and europium complex were carried out on a Nonius Kappa CCD single-crystal XRD-equipped Oxford Cryotream 700 (Oxford Cryosystems, UK). Thin–layer chromatography (TLC) films were visualized under UV light (254 nm) after treatment with iodine vapor, or heating treatment followed by exposure to 5% phosphor-molybdic acid in ethanol. Flash column chromatography was performed to purify all synthesized compounds using Merck (cat. no. 9385) 40–63 mm silica gel 60.

### 3.3. Synthesis of 2-(2-Chlorophenyl)-1-methyl-1*H*-imidazo[4,5-f][1,10]phenanthroline (Cl-MIP)

1,10-Phenanthroline-5,6-dione (phen-dione) was prepared as in a previous study [31]. 2-Chloroimidazophenanthroline (Cl-IP) was prepared by refluxing a mixture of phen-dione (0.53 g, 2.5 mmol), 2-chlorobenzaldehyde (0.50 g, 3.5 mmol), ammonium acetate (3.88 g, 50 mmol), and glacial acetic acid (7 mL) for 2 h in accordance with the Steck and Day method [42]. Chloro-phenylmethyl imidazophenanthroline (Cl-MIP) was synthesized through the methylation of Cl-IP, using sodium hydride in dry dimethyl-formamide (DMF) under N_2_ with EA: *n*-hexane of 20:1 elution to provide a favorable yield (85%) of brown powder. The melting point was 254–255 °C. ^1^H-NMR (500 MHz, CDCl3, 25 °C) *δ*: 9.21–9.15 (m, 2H), 9.05 (dd, 1H, *J* = 8.1, 8.2 Hz), 8.79 (dd, 1H, *J* = 8.2, 8.5 Hz), 7.75–7.66 (m, 3H), 7.60 (dd, 1H, *J* = 1.2, 8.0 Hz), 7.58–7.46 (m, 2H), 4.14 (s, 3H). ^13^C-NMR (125 MHz, CDCl_3_, 6 25 °C) *δ*: 151.0, 149.0, 148.0, 145.0, 144.2, 136.2, 134.8, 132.9, 131.7, 130.4, 129.9, 129.5, 128.1, 127.4, 125.5, 124.1, 123.6, 122.5, 120.2, 34.9. HRMS (ESI) calcd. For C_20_H_13_ClN_4_Na [(M+Na)^+^]: 367.0721, determined 367.0719.

### 3.4. Synthesis of Eu(DBM)_3_Cl-MIP Complex

The Eu(DBM)_3_Cl-MIP was synthesized using the following method. Dibenzoyl methane (DBM) at (1.36 g, 6 mmol) and Cl-MIP (0.74 g, 2 mmol) were dissolved in 15 mL of hot anhydrous ethanol. NaOH (1 N, 6 mL) was added, and the pH was adjusted to 7.0. The mixture was stirred while 2 mmoles of europium salt (EuCl_3_·6H_2_O) solution was added drop-wise. The mixture was then stirred for 5 h at 50 °C. After the mixture had cooled, a brown crude product was collected. Flash chromatography with EA: *n*-hexane at 20:1 provided Eu(DBM)_3_Cl-MIP (67% yield for crude) as a pale-yellow product. The melting point was 233–234 °C. ^1^H-NMR and ^13^C-NMR data of this compound are not shown here because of its magnetic property. The structure of Eu(DBM)_3_Cl-MIP confirmed by its single crystal data were shown in ESI^†^. HRMS (ESI) calcd. for C_65_H_46_ClEuN_4_NaO_6_ [(M+Na)^+^]: 1189.2210, determined 1189.2196.

### 3.5. Eu(DBM)_3_Cl-MIP/PBMA Composite Sample Results

A mixture containing Eu(DBM)_3_Cl-MIP, BMA monomer, and photoinitiator was dip-coated on a slide (2.54 cm × 4.0 cm × 1.01 mm), the obtained film thickness was between 22–24 μm (Scheme 1). The coated substrate was subsequently covered with polyethylene terephthalate (PET) film for UV light-irradiation under oxygen-free conditions. The coated composites were placed in a 400-W UV-light curing instrument (OPAS, TX-500, Taiwan) with a light source at 365 nm and an irradiation intensity of 50–70 mW/cm^2^. The distance between the coated substrate and light source was 8 cm. The content of Eu(DBM)_3_Cl-MIP ranged between 1.0–2.4 wt%, based on the monomer. The irradiation duration required to obtain the resultant products ranged from 250 to 400 s. The amount of UV-photoinitiator used was fixed at 3 wt% based on BMA. After the UV-irradiated sample had cooled to ambient temperature, the PET film was removed and immediately dried in a vacuum for 30 min. The film deposited on the substrate surface was transparent, colorless, and smooth with a thickness of 25–35 ± 5 μm. The Eu(DBM)_3_Cl-MIP of various contents and cured for different times was analyzed using photoluminescence (PL) and PL excitation (PLE) spectroscopy, performed using an F-4500 (Hitachi, Japan) equipped with a solid-sample holder and a 450-W Xe lamp. Ion-binding tests were stoichiometrically added to form a mixture of metal ions, including anhydrous FeCl_3_, MgCl_2_, CaCl_2_, and ZnCl_2_ dissolved in BMA (0.0243 g/0.5 g BMA) before UV irradiation.

### 3.6. Polymer Sample Results

Differential scanning calorimetric (DSC) measurements were carried out using a PerkinElmer 7 series thermal analyzer (PE, USA) at a heating rate of 10 °C/min, from room temperature to 200 °C. Thermal gravity analysis (TGA) was performed on an SDT Q600 V20.5 Build 15 Universal V4.4A (TA Instruments, USA), at a heating rate of 10 °C/min, from room temperature to 450 °C. Molecular weight distributions were determined using the Waters Alliance System (Waters 2414 differential refractive index detector, MA, USA), using a series of three linear styragel columns (HT2, HT4, and HT5) at ambient temperature; THF and 1 g/L of LiBr were used as the eluent. UV-visible absorption spectra were recorded on a Varian UV-Vis spectrometer (USA), using a quartz cuvette with 1-cm double-beam wavelength scanning. GPC was performed to yield an average molecular weight <*M*_n_> of 8775 and an average distributed molecular weight <*M*_w_/*M*_n_> of 2.38. The elution trace was approximately mono-modal and symmetrical, implying that the system created homogeneous photo-polymerization. Thermal stability tests revealed that the temperature of PBMA with a 5 wt% weight loss achieved 220 °C, and the maximum degradation temperature was 350 °C, which can be considered a suitable rank 1 used in the polymer matrix. In addition, this europium dopant had a melting point of 234 °C and *T*_g_ of 137 °C, indicating that the europium-composite was associated with favorable thermal stability for optical applications.

## 4. Conclusions

We reports the synthesis of a promising composite, consisting of a Eu(DBM)_3_Cl-MIP dispersed in a BMA monomer with subsequent UV-light polymerization. This synthesized Eu(DBM)_3_Cl-MIP complex, containing a new ligand of Cl-MIP, exhibited a larger absorption antenna function. It demonstrated a typical ternary europium narrow-band emission and long emission lifetime, but also demonstrated an absorption band response because of aromatic π–π stacking features. The aggregation tendency of the europium complex resulted in defect emissions and PL fluorescence decay. In this work, we employed PBMA as a dispersive matrix by conducting a finely controlled procedure. Furthermore, Eu^3+^ signals were enhanced by approximately 2.1-fold, as compared to the neat europium complex; whereas the defect emissions were suppressed by the PBMA matrix. In addition, the ratio between the pure Eu^3+^ and defect emissions achieved an enhancement factor of >two orders of magnitude. A series of ion-binding detects exhibited larger PL changes, especially for Fe^3+^-ion via chelating to PBMA, showed a more efficiently enhanced PL signal, and reached a 60% increase level, relative to the referenced sample. To conclude, this new europium composite demonstrated a promising application for optical detection materials.

## Supplementary Information



## Figures and Tables

**Table 1:** Product Compositions, Optical Absorption, Photoluminescence (PL), Quantum Yield, Electrochemical Data, and Thermal Properties.

Compound	λ_abs_^a^ (nm)	Band gap b (eV)	PL λ_max_ sol/film c	Φ d,e sol/film	LUMO/HOMO f (eV)	T_m_/T_g_^g^ (°C)
Cl-MIP	288	3.4	394	0.58/0.32	−2.40/−5.80	255/N.D
Eu(DBM)_3_Cl-MIP	294, 352	3.0	611	0.38/0.63	−2.98/−5.98	234/137
PBMA	257, 263	4.8	274	N.D h	N.D	N.D/47

poly-BMA = PBMA, Cl-MIP = chloro-phenylmethyl imidazophenanthroline, benzyl methacrylate = BMA, Dibenzoyl methane = DBM.

a In dichloromethane (*ca.* 6.0 × 10^−5^ M);

b From absorption onset values;

c Vaules obtained by solid sample holder;

d Cl-MIP in solution by using the referenced 2-(2,6-*di*-*tert*-butyl anthracen-9-yl)-1-methyl-1*H*-imidazo[4,5-f][1,10]phenanthroline had a quantum yield of 0.78 (Ref. [26]);

e Φ values for solid films were by integrating sphere method; Eu(DBM)_3_Cl-MIP in solution was by using 4-dicyano-methylene-2-methyl-6-*p*-dimethylaminostyrl-4*H*-pyan (DCM) in *n*-propanol (Φ = 57% ± 0.02%) (Ref. [35]);

f HOMO values were calculated from photoelectron analysis, while LUMO values were calculated by subtracting band gap values from their HOMO values;

g DSC traces with a heating rate of 10 °C/min in nitrogen atmosphere;

h No data.

**Table 2:** Emission Lifetime of Eu(DBM)_3_Cl-MIP with Different Structures.

Compound	*τ*_1_ (*a*_1_)	*τ*_2_ (*a*_2_)	Avg. lifetime, μs
Eu(DBM)_3_Cl-MIP	565 (0.625)	565 (0.300)	565
Eu(DBM)_3_Cl-MIP/PBMA	695 (0.648)	142 (0.276)	651
* Eu(DBM)_3_Cl-MIP/PBMA	505 (0.822)	1251 (0.143)	730

* Treatment with argon purging and freeze-thaw cycles.
