# Supplementary material for: Photosensized Controlling Benzyl Methacrylate-Based Matrix Enhanced Eu3+ Narrow-Band Emission for Fluorescence Applications
Source: Int J Mol Sci. 2012 Mar 21;13(3):3718–37. doi: 10.3390/ijms13033718 (PMC3317738; doi:10.3390/ijms13033718)
Supplement: Supplementary file 1 [file ijms-13-03718-s001.pdf]

Supporting Information

Figure S1. The molecular structure of Cl-MIP CH_2Cl_2, showing the atom-labeling scheme and thermal ellipsoids drawn at the 50% probability level.

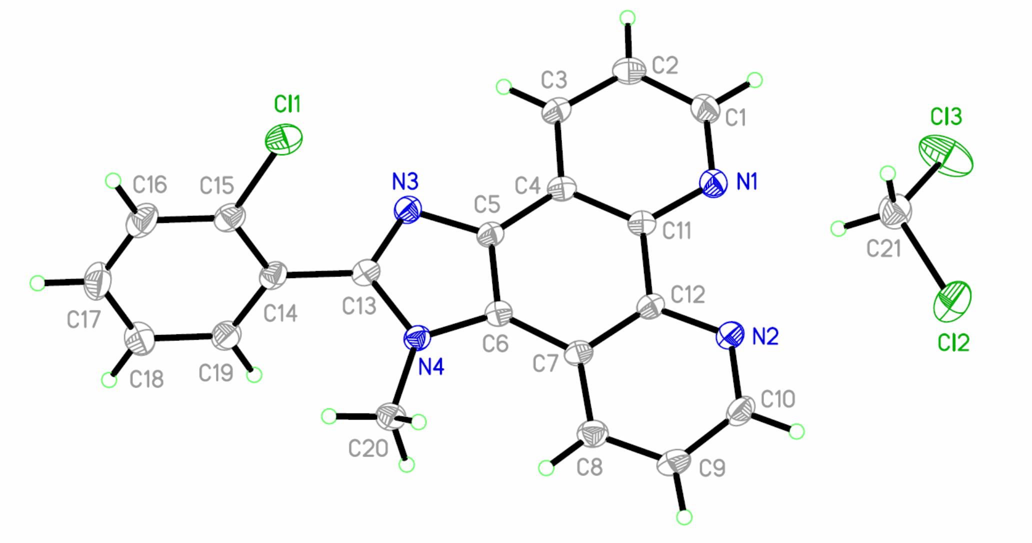

Figure S2. The packing of Cl-IP CH_2Cl_2 views with b-axis. Dashed lines represent the hydrogen bonding.

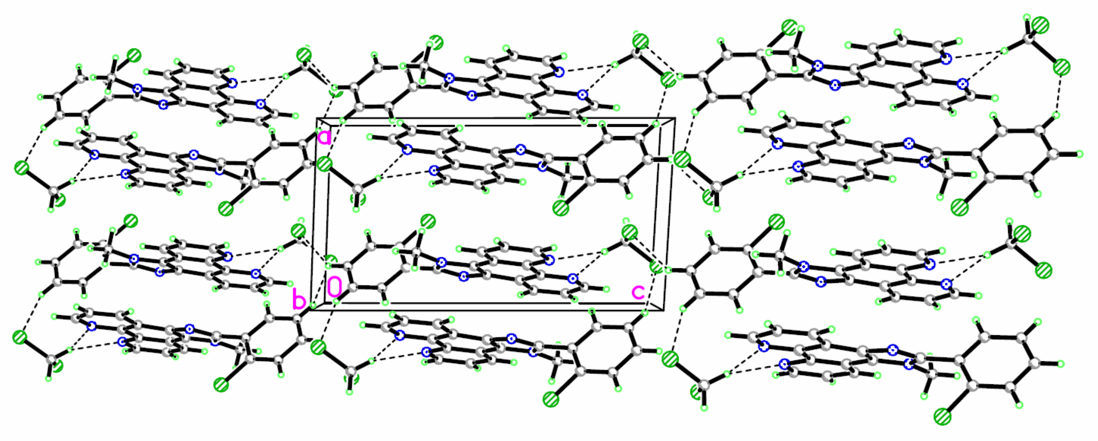

Table S1. Hydrogen Bonds for Cl-MIP [Å & angle (degree)].

D-H ··A	d(D-H)	d(H . . . A)	d(D . . . A)	<(DHA)
C(21)-H(21B) ··N(1)	0.99	2.38	3.241(3)	145.1
C(21)-H(21B) ··N(2)	0.99	2.42	3.286(3)	145.9
C(17)-H(17) ··Cl(2)#1	0.95	2.82	3.588(2)	138.5
C(18)-H(18) ··Cl(3)#2	0.95	2.82	3.629(2)	144.1

Symmetry transformations used to generate equivalent atoms: #1 x, y−1, z−1, #2 -x, −y+1, −z+1

Table S2. Crystal data and structure refinement for Cl-MIP·CH_2Cl_2

Identification code	Cl-MIP·CH_2Cl_2	
Empirical formula	C_{21} H_{15} Cl_3 N_4	
Formula weight	429.72	
Temperature	150(2) K	
Wavelength	0.71073 Å	
Crystal system	Triclinic	
Space group	P-1	
	a = 7.8827(5) Å	=89.3447(14)°.
Unit cell dimensions	b = 8.7562(6) Å	= 87.3009(15)°.
	c = 14.0231(9) Å	= 79.1288(14)°.
Volume	949.48(11) $Å^3$	
Z	2	
Density (calculated)	1.503 Mg/m^3	
Absorption coefficient	0.498 mm^{-1}	
F(000)	440	
Crystal size	0.40 × 0.38 × 0.14 mm^3	
Theta range for data collection	1.45 to 27.50°.	
Index ranges	−10 <= *h* <= 10, −11 <= *k* <= 11, −18 <= 1 <= 18	
Reflections collected	12602	
Independent reflections	4349 [R(int) = 0.0311]	
Completeness to theta = 27.50°	99.3%	
Absorption correction	Semi-empirical from equivalents	
Max. and min. transmission	0.9336 and 0.8257	
Refinement method	Full-matrix least-squares on F^2	
Data / restraints / parameters	4349 / 0 / 254	
Goodness-of-fit on F^2	1.116	
Final R indices [I>2sigma(I)]	R1 = 0.0427, wR2 = 0.1112	
R indices (all data)	R1 = 0.0508, wR2 = 0.1227	
Largest diff. peak and hole	0.616 and −0.552 e.$Å^{-3}$	

Table S3. Crystal data and structure refinement for Eu(dbm)$_3$Cl-MIP$\cdot$CH$_2$Cl$_2$.

Identification code	Eu(dbm)$_3$Cl-MIP$\cdot$CH$_2$Cl$_2$
Empirical formula	C_{66} $H_{48}Cl_3Eu$ N_4O_6
Formula weight	1251.39
Temperature	150(2) K
Wavelength	0.71073 Å
Crystal system	Monoclinic
Space group	P2(1)/c
Unit cell dimensions	a = 12.2225(6) Å = 90 °.
	b = 20.7889(10) Å =96.3883(10) °.
	c = 22.1103(11) Å = 90 °.
Volume	5583.2(5) Å^3
Z	4
Density (calculated)	1.489 Mg/m^3
Absorption coefficient	1.325 mm^{-1}
F(000)	2536
Crystal size	0.40 × 0.25 × 0.25 mm^3
Theta range for data collection	1.35 to 27.50 °.
Index ranges	−15<= *h* <= 15, −25 <= *k* <= 27,
	−28 <= l <= 28
Reflections collected	42634
Independent reflections	12815 [R(int) = 0.0331]
Completeness to theta = 27.50 °	99.9%
Absorption correction	Semi-empirical from equivalents
Max. and min. transmission	0.7329 and 0.6192
Refinement method	Full-matrix least-squares on F^2
Data / restraints / parameters	12815 / 0 / 722
Goodness-of-fit on F^2	1.055
Final R indices [I>2sigma(I)]	R1 = 0.0348, wR2 = 0.0790
R indices (all data)	R1 = 0.0421, wR2 = 0.0831
Largest diff. peak and hole	1.096 and -0.971 e.Å^{-3}

© 2012 by the authors; licensee MDPI, Basel, Switzerland. This article is an open access article distributed under the terms and conditions of the Creative Commons Attribution license (http://creativecommons.org/licenses/by/3.0/).
